# A study of guidelines for respiratory tract infections and their references from Swedish GPs: a qualitative analysis

**DOI:** 10.1080/02813432.2020.1717073

**Published:** 2020-02-07

**Authors:** M. Tyrstrup, M. André, A. Brorsson, H. Gröndal, E.-L. Strandberg, K. Hedin

**Affiliations:** a Department of Clinical Sciences, Family Medicine, Lund University, Malmö, Sweden;; b Department of Public Health and Caring Sciences, Family Medicine and Preventive Medicine, Uppsala University, Uppsala, Sweden;; c Department of Medicine and Health Sciences, Family Medicine, Linköping University, Linköping, Sweden;; d Center for Primary Health Care Research, Malmö, Sweden;; e Department of Sociology, Uppsala University, Uppsala, Sweden;; f Fururum, Jönköping, Sweden;; g Department of Medical and Health Sciences, Linköping University, Linköping, Sweden

**Keywords:** Adherence to guidelines, antibiotic prescribing, general practice, national guidelines, respiratory tract infections

## Abstract

**Background:** National guidelines are important instruments in reducing inappropriate antibiotic prescriptions. Low adherence to guidelines is an acknowledged problem that needs to be addressed.

**Method:** We evaluated established characteristics for guidelines in the guidelines for lower respiratory tract infection, acute otitis media and pharyngotonsillitis in primary care. We studied how doctors used these guidelines by analysing interviews with 29 general practitioners (GPs) in Sweden.

**Results:** We found important between-guidelines differences, which we believe affects adherence. The GPs reported persistent preconceptions about diagnosis and treatment, which we believe reduces their adherence to the guidelines.

**Conclusion:** To increase adherence, it is important to consider doctors’ preconceptions when creating new guidelines.

## Background

Antibacterial agents are essential in human medicine [1], but inappropriate use of antibiotics has resulted in increased bacterial resistance [2,3]. Primary care is an important target for reducing antibiotic consumption, since 90% of antibiotics used in Sweden are prescribed in outpatient care, of which about 60% are prescribed by general practitioners (GPs). Most antibiotics are prescribed for respiratory tract infections (RTIs) [4,5]. Large variations in antibiotic prescriptions between countries, counties, primary health care centres (PHCCs) and prescribers suggests that antibiotics are overprescribed [4–8].

Many factors influence the complex process of prescribing antibiotics [9,10]. Multiple interventions including educational measures for physicians have been shown to improve antibiotic prescribing [11]. As part of the Swedish strategy to curb antibiotic use, national evidence-based guidelines for different infectious diagnoses have been developed by the Public Health Agency of Sweden and the Swedish Strategic Programme Against Antibiotic Resistance (Strama) [12]. Strama is a national organization with local groups in each county working to preserve the effectiveness of antibiotics. Part of Strama’s strategy is to provide feedback to prescribers and to implement and review national guidelines for diagnoses and treatment. The evidence-based guidelines recommend antibiotic treatment only when studies have shown patient benefit. This contrasts with the earlier practice of identifying a bacterial infection to be treated with antibiotics. The national guidelines are developed in collaboration with physicians in hospital and primary care and finalized through consensus [13]. The guidelines are implemented in several ways and introduced at most PHCCs in Sweden by outreach visits by GPs and pharmacists representing Strama and feedback on antibiotic prescriptions. Studies confirm that Swedish GPs are generally well aware of the guidelines [14].

Improved adherence to the national guidelines for lower respiratory tract infections (LRTIs), acute otitis media (AOM) and pharyngotonsillitis has been noted recently, but the remaining variability in antibiotic prescription rates between prescribers indicates some continuing non-adherence [5].

Guideline implementation is a complex process with many influencing factors, barriers and facilitators that have been explored and discussed [15]. Two decades ago, specific characteristics of guidelines were identified as influential on adherence [16–19]. In a study of primary care in the Netherlands, Grol et al. [17] identified and related attributes of guidelines to performance data. Seven attributes concerned the guideline itself (intrinsic factors) and the importance of these attributes was corroborated in a later study [19]. Better compliance was found to correlate with guidelines with (1) concrete description of the desired performance, (2) specific (vs. vague) recommendations, and (3) with low complexity. Better compliance was also found to correlate with (4) compatibility with existing norms and values, (5) no new competence or skills needed, (6) no consequences on practice management (extra resources) and (7) no necessary changes to routines and habits. Lately, such intrinsic factors of guidelines have been discussed in the international AGREE enterprise, which aims to increase the quality and implementability of guidelines [20] In a recent study family physicians emphasized the importance of guidelines being simple, clear, uncomplicated and easy to use [21].

Few studies from primary care, however, have explored the specific characteristics of guidelines in relation to GPs’ adherence [22]. The Swedish guidelines for LRTI, AOM and acute pharyngotonsillitis (tonsillitis) were similar in their development and implementation, but vary in their recommended measures and concrete descriptions, which might affect adherence. The aim of the current study, therefore, was to explore GPs’ stated management of patients with cough, earache, and sore throat and their adherence to guidelines and to explore how these related to the specific characteristics of each guideline.

## Method

We chose a qualitative approach to explore GPs’ stated adherence to three different guidelines for LRTI, AOM and acute tonsillitis in relation to the characteristics of those guidelines. In the first of two steps we reviewed the current guidelines for the attributes associated in the literature with guideline compliance and then analysed semi-structured interviews with GPs asking how they used the guidelines in a typical clinical case. The interviews with GPs were conducted as part of a larger mixed method study exploring factors influencing antibiotic prescribing [14].

In the second step of the process, we combined our findings from the guideline review and interview analyses and explored how GPs stated use of a guideline was associated with its specific characteristics.

### Data collection and analysis

#### Review of guidelines

The characteristics of the guidelines for LRTIs, AOM and tonsillitis were reviewed using a template analysis [23] of six of the seven intrinsic attributes associated with compliance identified by Grol et al. [17]. These attributes are: concrete descriptions of the desired performance; vague and unspecific recommendations; complexity (complex decision trees with many different elements); new competence or skill needed; consequences for practice management (extra resources); and changes to routines and habits needed. The attribute ‘compatibility to existing norms and values’ could not be evaluated by analysing the guidelines, but was instead evaluated through the interviews. Three of the researchers (MT, KH and MA) determined independently whether each attribute was present in the studied guidelines. Disagreement was resolved in discussion until consensus was achieved.

A translated short version of the national guidelines is provided in Supplement 1.

#### GP interviews: methods and analysis

During January and February 2014, a strategic sample of 29 GPs from 8 PHCCs in 3 different counties in Sweden were interviewed. The GPs represented a variety of gender, age, educational background, work experience, urban/rural location of the PHCCs and areas with high and low antibiotic prescribing. All participating PHCCs were publicly run. All GPs who were invited to be interviewed agreed to participate.

The purpose of the interviews was to explore GPs’ stated management of, and adherence to national guidelines for, patients with cough, earache, and sore throat. Individual semi-structured interviews with open-ended questions ensured the interviewees’ own narratives were collected. Rather than the GPs’ views on the outline of the guidelines, we were interested in how they described their case management in light of the guidelines and what they found problematic. A translated interview topic guide is provided in Supplement 2. Four researchers were GPs and two were social scientists. The interviews were audio recorded, professionally transcribed and translated word-for-word to avoid altering GPs’ meaning.

The interviews were analysed inductively using systematic text condensation [24]. We identified themes relevant for adherence/non-adherence to guidelines.

In the second step, the themes resulting from the analysis of the interviews were compared with the attributes defined by Grol et al. to which they corresponded. This part of the analysis was an iterative process of several meetings for discussion amongst the researchers until consensus was reached. The analysis was performed manually.

## Results

The analysis of the guidelines showed differences in the attributes of the three (Table 1). The guideline for tonsillitis was the most concrete and had the most detailed descriptions of the desired performance, while the guideline for LRTI was more vague. The guideline for AOM was the most complicated and the only one requiring some new equipment and new skill.

**Table 1.** Characteristics for diagnosis of lower respiratory tract infections (LRTI), acute otitis media (AOM) and tonsillitis according to evidence in the literature about guidelines (Grol et al. [17]).

	Presence of characteristics		
	LRTI	AOM	Tonsillitis
Concrete and precise description of desired performance	No	Yes	Yes
Vague and not specific	Yes	No	No
Complex (composed of many different elements, contains a complex decision tree or conditional factors)	No	Yes	No
Demands acquisition of new competence or skill	No	Yes	No
Consequences for practice management (new equipment)	No	Yes	No
Demands changing routines and habits	No	Yes	No

Demographic characteristics of the 29 participants are presented in Table 2.

**Table 2.** Description of the 29 interview participants.

Category	Variable	Proportion of participants
Age	<45	24% (7/29)
Years of experience as a general practitioner	>10	59% (17/29)
Gender	Female	45% (13/29)
Medical education	In Sweden	66% (19/29)
Location of practice	City	28% (8/29)
	Town	55% (16/29)
	Village	17% (5/29)
Antibiotic prescription level in the county	High	45% (13/29)
	Medium	10% (3/29)
	Low	45% (13/29)

In the inductive analysis of the interviews four themes were found relevant to stated adherence to guidelines. The themes ‘clinical skills’ and ‘organizational flow’ corresponded to the attributes ‘consequences for practice management’ and ‘changes to routines and habits’. The theme ‘persisting concepts’ corresponded to the attribute ‘conformity to existing norms and values’. The statements by the GPs in the theme ‘guideline knowledge’ corresponded to ‘concrete description of desired performance’, ‘vague recommendations’ and ‘complexity of decision-making’.

### Concrete or vague descriptions and complexity of decision-making

#### Analysis of the guidelines

The analyses of the guidelines showed that the guideline for tonsillitis had the most concrete and detailed description presented in a flowchart (Table 1). This was also the guideline that required the least complex decision making. The guideline for LRTI was assessed as the opposite: It was the vaguest guideline with fewest detailed descriptions. The guideline for AOM was quite detailed and concrete, but it also contained a complicated message with many different elements. The guideline for tonsillitis was therefore found to be most likely to be followed.

#### Analysis of the interviews

All GPs expressed knowledge about all three guidelines, but in their reported management of the three sample patient cases their stated adherence varied according to the guideline.

The guidelines for LRTI seemed to be well-known and generally well followed. In discussing LRTIs, the GPs focused on identifying pneumonia from a wide range of differential diagnoses, in line with the guideline. Despite the vaguely described and rather complex diagnostic procedure, the GPs reported no problem diagnosing pneumonia. However, the guideline’s concrete recommendation to use O_2_ saturation and breathing rate in the diagnosis was not commonly addressed in the interviews.

For patients with earache, in line with the guideline, the GPs focused on visually assessing the eardrum. However, they seldom mentioned the importance of assessing eardrum mobility, which the guideline states is important. Several GPs mentioned that the guideline applied to patients of certain ages, but had difficulty remembering which ages. Others were not aware that age was part of the criteria for treatment.

Interview 28, page 11 (Quotation A)

Interviewer (I): Mmm. And what do you do, which patients do you give antibiotics to then?

GP28: All those where I think it looks nasty, like otitis.

I: On the eardrum?

GP28: Yes, it’s the eardrum I assess.

I: Mmm. But you… you don’t take into consideration how old the children are?

GP28: No, I don’t think so.

The guideline was described as complicated and therefore difficult to internalize and follow.

The guideline on detecting Group A streptococcus (GAS)-induced tonsillitis requires at least three Centor criteria for conducting a rapid antigen detection test (RADT) and recommends antibiotics only if the RADT is positive for GAS. Despite these concrete descriptions of the desired performance, many GPs mentioned only one or two Centor criteria. Thus, the criteria gave them no clear guidance on the use of RADT, which contrary to the guideline, they used when suspicion of tonsillitis was low and only a few criteria present. The GPs also reported that clinical findings could overrule the guideline recommendation on when to use RADT.

Interview 10, page 12 (Quotation K)

GP10: And… if I have met a patient who fits the criteria and if I have found something objective and precise… I… the most important thing for me is the clinical status.… How the patient is feeling, is affected… and so if I find something by objective examination, when I look at the throat, that… for example, I take no samples, I treat directly.

A negative RADT for GAS was seldom mentioned as a reason not to prescribe antibiotics.

### Need for new competence, consequences for practice management and demands to change routines and habits

#### Analysis of the guidelines

Only the guideline for AOM contained recommendations requiring some new skills in conjunction with new equipment, which in turn potentially called for organizational change. The guideline recommends eardrum mobility be assessed through pneumatic otoscopy, ear microscopy or tympanometry. The guidelines for LRTI and tonsillitis called for no new skills or organizational change.

#### Analysis of the interviews

From the interviews, we found the recommended diagnostic tools for diagnosing AOM were rarely used. Several GPs’ offices lacked pneumatic otoscopy equipment; the ear microscope or tympanometry equipment were located in a special room in the health centre. The GPs, however, preferred to stay in the examining room for the whole consultation due both to habit and lack of time, and not all were skilled in the use of the equipment. Lack of skill and aversion to organizational change thus hampered adherence.

### Compatibility with existing norms and values

#### Analysis of the interviews

For patients with LRTI and tonsillitis, many GPs talked about the importance of differentiating between viral or bacterial conditions to decide who needed antibiotics, suggesting a norm that bacteria always needs to be identified and treated. Near-patient C-reactive protein (CRP) test was regarded by the GPs an important tool to differentiate between bacteria and virus. The use of CRP test is in line with the guidelines for LRTI, but not for tonsillitis.

In accordance with the guideline for LRTI, CRP was tested early in the clinical assessment.

Interview 8, page 15 (Quotation M)

GP8: I usually take CRP for safety’s sake and if necessary I prescribe antibiotics then.

The diagnosis of acute bronchitis was associated with a viral origin and pneumonia with bacterial, corresponding to the result of the CRP tests.

Interview 10, page 24 (Quotation R)

GP10: Yes, if I have a patient with a cough and if I suspect infection… I take CRP… I first want to see what exactly it looks like… it can be that you have a cough… but the inflammation… it’s viral inflammation.… So it’s not completely certain that it’s bacteria.… So CRP… sometimes it helps us because you… well, it can be…bacterial that’s needed…

In patients with otitis media, however, almost none mentioned the need to differentiate between viral and bacterial conditions. The crucial point stated by the GPs was to identify and diagnose a condition with red eardrum as AOM, not mentioning any judgement of the mobility of the eardrum or other factors which are important for the diagnosis. Most GPs also seemed to have accepted the self-healing character of bacterial AOM and did not worry much about complications. GPs stated the guidelines for AOM did not conflict with their existing values.

For many GPs, the guidelines for acute tonsillitis did not seem to be compatible with existing values. For example, many GPs did not appear to believe that tonsillitis is a self-healing condition. Instead they had great trust in using antibiotics to prevent complications, in contrast to the guideline statement that tonsillitis is treated to shorten symptom duration. Some GPs said they differentiated between virus and bacteria through the clinical gaze instead of using the recommended Centor criteria.

Interview 8, pages 22–23 (Quotation S)

GP8: If it (GAS test) is negative and the clinical picture indicates bacterial infection… and the patient has a temperature, if the patient feels tired, if the patient has difficulty swallowing… I give antibiotics.

Others told of using the non-recommended CRP. CRP seemed to be regarded as a superior test that could confirm or exclude significant bacterial disease.

Interview 10, page 14 (Quotation O)

I: When do you test CRP?

GP10: When you… when I’m uncertain about…

I: Uncertainty?

GP10: Uncertain whether it is tonsillitis or just a cold or something like that, those patients who don’t meet the criteria, or they meet one or two of these criteria, then… you’re uncertain whether you can see anything in the throat so you take samples to confirm this.

I: Mmm. Confirm, but perhaps also… also when you want to exclude something?

GP10: To exclude, yes.

GPs also mentioned patients asking for RADT testing regardless of Centor criteria.

Interview 15, page 15 (Quotation Z)

GP15: And… sometimes patients come just to… they want to take a throat sample.… So then the sample is more some form of… well… a way to make the patient secure and satisfied, that’s how it is.

I: Mmm.

GP15: But… I understand that too, that in… in the long run it would have been better to devote a little time and perhaps explain why you shouldn’t take this sample, but the thing is how little… time you have and what contact you think you get with the patient.

Compatibility with existing norms thus varied between the guidelines and conflict between guidelines and existing norms seemed most pronounced for tonsillitis.

### Interference with GPs’ decision making, compatibility with existing norms and adherence to guidelines

#### Analysis of the guidelines and the interviews

We noted that concrete descriptions may interfere with GPs’ decision making to various degrees. There is more room for GPs’ idiosyncratic interpretations when the diagnostic process is not transparent but includes elements exclusively performed and interpreted by the doctor, for example examination of the eardrum or auscultation of the lungs. Less room for interpretation is given when tests or other standard measures are used to validate the diagnostic process, and this characterizes the guideline for tonsillitis well.

**Figure 1.**
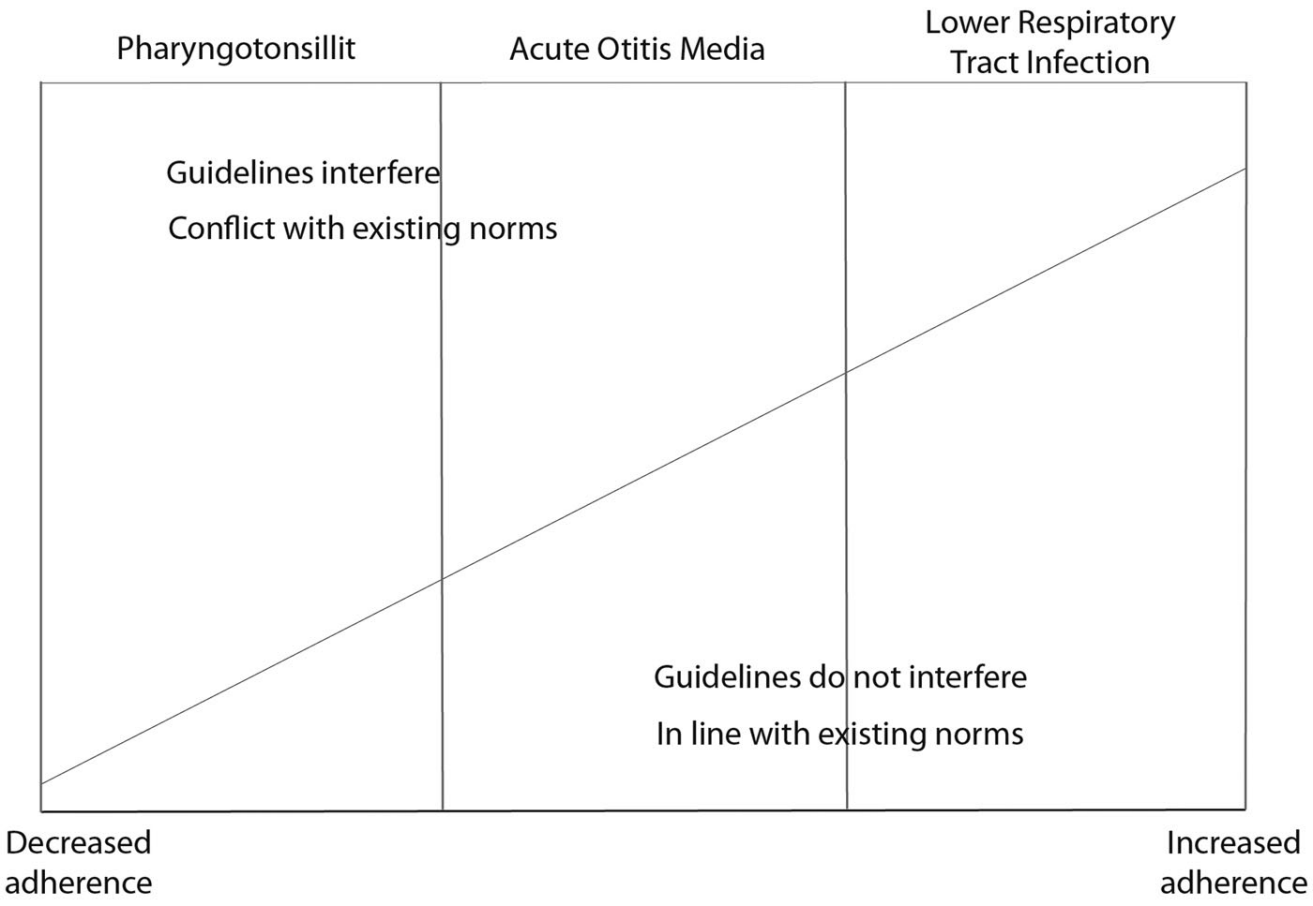
Level of guideline interference with existing norms.

We found that if the recommendation in the guidelines was not compatible with existing norms, adherence was low regardless of other attributes (Figure 1). This was most pronounced for tonsillitis. The low adherence to the tonsillitis guideline seemed to be exacerbated by the constraint on GPs’ decision making by the recommendation to use the Centor criteria and the near-patient RADT. Non-adherence to the AOM guideline was likely attributable to GPs inability or unwillingness to perform a diagnostic mobility test of the eardrum and not correctly remembering the recommendations regarding indications for antibiotic treatment. Also, given that visual examinations of the ear cannot be reviewed by anyone other than the examiner, they risk being unduly subjective. Few exceptions to the LRTI guidelines were reported and CRP testing was in line with existing norms.

## Discussion

### Main findings

This qualitative study analysed important attributes of guidelines affecting adherence and GPs’ management of patients with cough, earache and sore throat in relation to the national guidelines. The studied attributes differed between the guidelines. Despite implications of previous research, the guideline for tonsillitis seemed to be the least adhered to, even though it fulfilled most of the attributes identified as important for adherence. The opposite was found for the guideline for LRTIs. The crucial attribute for adherence overall seemed to be compatibility with existing norms.

If recommendations in the guidelines are not compatible with GPs’ existing norms, adherence will be low even if they have all of the important attributes important for adherence. This effect might be reinforced by perceived interference in the GPs’ decision making.

### Strength and limitations of the study

We chose the attributes for adherence identified by Grol et al. [17] because although the study is almost 20 years old, it is still frequently cited. All three guidelines analysed were developed and distributed through the same process with only four years’ difference. In this process two of Brouwers’ three key principles of importance for implementability, credible and representative developers of guideline content and high-quality synthesis and contextualization of evidence, were comparable [25,26]. The third principle, optimal use of language and format to convey the recommendations, however, differed between the three guidelines [25,26]. Moreover, all three guidelines had been implemented similarly by outreach visits from Strama to the PHCCs. These baseline conditions make it possible to compare the significance of the intrinsic attributes of the guidelines.

The strength of the interview study was the strategically selected GPs participating, representing both sexes and different ages, educational backgrounds, and regions of Sweden. The result should therefore be transferable to the population of Swedish GPs. Interviews were semi-structured and performed according to an interview guide to ensure dependability. However, because semi-structured interviews are not as in-depth as interviews with open questions, the information gathered may have been limited. Five different researchers performed the interviews and four of them participated in the analysis, which increased the credibility of the data and added different perspectives.

In the inductive analysis of the interviews, the attributes established earlier by Grol et al. [17] were found to be relevant. The themes from the interview study were found to correspond well enough for a further analysis, although with different labels.

The study relies only on what GPs report they do, which may or may not correspond to their actual performance, for which we have no data. However, our findings are in line with earlier studies of performance in Swedish primary care [14,27].

### Findings in relation to previous studies

Recent studies have highlighted the importance of various attributes or characteristics of guidelines for adherence. Thus the format and language of guidelines have been added as important features [25,26]. Improvement in the way the guidelines are written may be a simple and cost-effective way to enhance adherence [26].

#### Compatibility with existing norms

The main message in all guidelines concerned the diagnostic process. Studies on infections in primary care show an evolving paradigm shift in recent decades from detecting and treating all bacterial infections to using antibiotics only for conditions where there is evidence the patient will benefit from treatment. This new paradigm, however, was reflected differently in the three guidelines in this study.

GPs stated the need to identify the infections as bacterial or viral in patients with LRTI and tonsillitis, but not in patients with AOM. The use of the near-patient CRP was often considered an important tool for patients with tonsillitis, and CRP results in patients with LRTI were thought usually to be consistent with the guidelines and diagnoses of acute bronchitis or pneumonia. Studies have shown that CRP testing facilitated adequate antibiotic prescribing [28], which for patients with acute bronchitis decreased by half between 2008 and 2013 in Sweden [5]. In contrast, for patients with tonsillitis the result of the CRP test reinforced the suspicion of an unspecified bacterial infection, which could overrule the use of the recommended Centor criteria and RADT [27].

The evidence that most tonsillitis in primary care is self-healing does not seem to have spread in Sweden. Patients are also used to, and might even demand, a RADT regardless of the Centor criteria. Studies of performance data from primary care in Sweden have confirmed non-adherent use of RADT and CRP [27,29]. The consultation rate for the diagnosis of tonsillitis in Sweden is twice that in the Netherlands and the antibiotic prescription rate three times higher [30]. Thus the idea that a sore throat needs consultation and treatment is probably widespread both in the population and among GPs in Sweden.

Although the guideline for AOM was described as complicated, the message that AOM is a self-healing condition regardless of aetiological agent seemed to have disseminated to both GPs and public. Consultation rates decreased by almost half between 2008 and 2013, explained not only by the introduction of the pneumococcal vaccine but also by better adherence to guidelines [5].

With a vague guideline non-adherence is more difficult to discover and the guideline for LRTI had few reported exceptions. Guidelines are drawn up to bring about change or to create uniformity, which is why vague guidelines have a lower potential to guide behaviour [18,26]. Likewise, when the diagnostic process includes elements performed exclusively by the doctor with no possibility of review (e.g. examination of the eardrum or auscultation of the lungs), even a concrete description may allow idiosyncratic interpretations. Among the studied guidelines this was most obvious for AOM, where the diagnosis is made exclusively by the GP. The guideline for tonsillitis was the opposite, calling for validation of the diagnosis with RADT.

Earlier studies have shown that guidelines that contradict common preconceptions and have low compatibility with existing norms risk not being accepted [18]. Although the guideline for tonsillitis was the most concrete and detailed of the three studied, it was still the least adhered to. Our interpretation is that compatibility with existing norms is the decisive factor in adherence.

#### New equipment or need for organizational change

The study also indicates the importance of taking consequences into account when recommending new equipment or need for organizational change. Non-adherence was quite common in the GPs’ description of management of patients with AOM, as they lacked both the equipment and skill to assess mobility of the eardrum. If new equipment or organizational changes are needed, GPs in Sweden need to rely on PHCC managers to make those changes possible.

Near-patient tests decrease both uncertainty and antibiotic prescribing and thus increase adherence to guidelines. RADT and CRP have been used in Swedish primary care for 30 years in managing patients with RTI. For patients with tonsillitis the results of RADT are not fully trusted, but the non-recommended CRP is used as a superior test in line with the outdated idea that a bacterial infection should be detected and treated [27]. Sweden is a low-prescribing country, but studies indicate that CRP in this context may increase antibiotic prescribing [27,31].

#### Implications

A survey of current norms and values among end users is not currently included in Swedish guidelines development. The results of this study suggest that exploring persistent conceptions and addressing them in guideline outlines may be key to their successful implementation [21]. Understanding and considering patients’ preferences in developing guidelines may also be a way to increase adherence [32]. These measures require awareness and additional time and resources for and from the guideline community and guideline writers. Primary care physicians may also be engaged in assessing the language and format attributes of recommendations and improving these according to their preferences [21].

The implementation programme for guidelines less compatible with existing norms needs to be more thorough. GPs should be alerted to the evidence supporting the recommendations, but education and conventional knowledge translation may be insufficient. Facilitating reflective consideration, individually and collectively, of how to perform in particular cases and situations may influence personal practice through recognizing practical wisdom and case knowledge [33]. More attention should be given during the outreach visits by Strama GPs and pharmacists to discussing controversial recommendations in peer review groups [34]. Particularly, the guidelines for tonsillitis need attention as they have caused concern among hospital doctors about complications [35]. The exactness of the guidelines for tonsillitis and their interference with GPs’ ability to improvise might make other types of health care providers such as nurses more suitable for patients with tonsillitis. Or perhaps the guidelines need to be rewritten to address persistent ideas. Tailored implementation strategies based on the identified barriers [36] may also be important for a successful implementation [37].

## Conclusion

We analysed interviews about GPs’ experiences diagnosing AOM, LRTIs and pharyngotonsillitis in relation to national guidelines. We found that if guidelines are not compatible with the GP’s existing norms, adherence might be low even if they display all the attributes found in the literature to be important for adherence. GPs’ persisting conceptions about diagnostic procedures and treatments are therefore important to address when revising and creating new guidelines, as they could otherwise act as a barrier to adherence.

## Supplementary Material

Supplemental Material

Supplemental Material

## Data Availability

Since sharing of data was not included in the approval from the ethics committee or the informed consent of participants, data will not be made public.
