# Supplementary material for: A study of guidelines for respiratory tract infections and their references from Swedish GPs: a qualitative analysis
Source: Scand J Prim Health Care. 2020 Feb 7;38(1):83–91. doi: 10.1080/02813432.2020.1717073 (PMC7054917; doi:10.1080/02813432.2020.1717073)
Supplement: Supplemental Material [file IPRI_A_1717073_SM7123.doc]

**Supplement 2.** Questions in semi-structured interviews, regarding three typical cases of earache, cough and sore throat.

**Child with earache**

What do you think it might be? How do you find out?

How do you normally examine/what techniques do you use?

What examination findings or symptoms lead you to prescribe antibiotics?

Which antibiotic do you normally use?

When do you use amoxicillin (Amimox)?

Do you use prescriptions in reserve?

When do you not prescribe antibiotics?

How is a new consultation time made available in the event of deterioration or no improvement?

What are you afraid might happen if you do not prescribe antibiotics?

**Patient with recent cough, a healthy middle-aged person**

What do you think it might be? How do you find out?

How do you use CRP?

How do you assess CRP?

What is your CRP limit (for diagnosis of pneumonia) for prescribing antibiotics)?

Is there something special in the anamnesis or status that leads you to prescribe antibiotics?

Which antibiotic do you normally use?

When do you use broader-spectrum antibiotics such as tetracyklin?

Do you use prescriptions in reserve?

When do you not prescribe antibiotics?

What are you afraid might happen if you do not prescribe antibiotics?

**Patient with sore throat**

What do you think it might be? How do you find out?

When do you take StrepA?

Do you trust the test?

How do you handle negative StrepA?

When do you take CRP in a patient with a sore throat?

What do you look for/how do you relate to the answer?

When do you take a culture in a patient with a sore throat?

What do you look for/what do you think?

What are you afraid might happen if you do not prescribe antibiotics?
