# Supplementary material for: A study of guidelines for respiratory tract infections and their references from Swedish GPs: a qualitative analysis
Source: Scand J Prim Health Care. 2020 Feb 7;38(1):83–91. doi: 10.1080/02813432.2020.1717073 (PMC7054917; doi:10.1080/02813432.2020.1717073)
Supplement: Supplemental Material [file IPRI_A_1717073_SM7115.doc]

**Supplement 1**. Short version of the national guidelines for acute otitis media, lower respiratory tract infections and pharyngotonsillitis.

**Acute otitis media (AOM)**

*Background:*

Acute otitis media, AOM, in children usually heals well without antibiotic treatment. Serious complications such as mastoiditis are uncommon. Antibiotic treatment is decreasing, but it does not exclude the risk of mastoiditis.

*Diagnostic criteria*:

Quick onset of symptoms, for example earache, crying, irritability, fever, impaired activity/appetite/sleep, usually during ongoing URTI.

Finding eardrum inflammation and pus in the middle ear or auditory meatus.

*Diagnostic aids:*

Use pneumatic otoscopy by means of Siegle’s speculum (otoscope with airtight head, magnifying glass and bulb) or otomicroscopy, preferably in combination with tympanometry.

Assessment of clinical findings when there are symptoms of AOM:

Certain AOM:

Purulent secretion and/or perforated/scarred eardrum, or bulging, non-transparent, changed colour, immobile eardrum

Uncertain AOM:

Non-transparent, changed colour, immobile, non-bulging eardrum, or the eardrum cannot be assessed

Not AOM:

Changed colour, mobile eardrum or transparent, retracted or in normal position, immobile eardrum (= SOM)

*Measures to be taken:*

Assessment by a doctor should be offered within 24 hours. There are rarely medical reasons for an evening or night examination. If the trouble disappears during the waiting time, no examination is necessary. Offer painkillers. Recommend elevated position.

At doctor consultations information should be provided about the normal course and any complications to look out for.

Patient information can be found e.g. at the Public Health Authority website. Recommend a new doctor consultation after 2–3 days if no or dubious improvement and immediately if it gets worse, regardless of whether antibiotic treatment is given or not.

If general health is affected (for example torpor, incapacity for normal contact or laughing and smiling, irritability) or the slightest sign of mastoiditis (redness, swelling, tenderness behind the ear or protruding outer ear) the patient should be examined at once and referred acutely to an ENT specialist or paediatric emergency clinic.

Active wait and see is recommended for:

- children aged 1–12 with certain AOM without complicating factors*
- patients regardless of age with uncertain AOM without complicating factors*.

If it is certain AOM, antibiotic treatment is recommended for:

- children aged 1–12 with AOM and complicating factors*
- children < 1 year and > 12 years and adults
- children < 2 years with bilateral AOM and all with perforated AOM regardless of age.

*Complicating factors in AOM

– Severe pain despite adequate analgesic treatment

– Sensitivity to infection due to other simultaneous disease/syndrome or treatment

– Deformities in the facial skeleton or inner ear

– Status after skull or face fracture

– Cochlea implant

– Known disease of the middle ear or previous ear operation (not plastic tube)

– Known sensorineural hearing impairment

**Acute bronchitis and pneumonia in adults (LRTI)**

*General advice:*

Always advise smokers to stop smoking in connection with respiratory tract infection.

If cough lasts > 4–6 weeks, consider spirometry and lung X-ray.

Unaffected patient with cough without known COPD, chronic bronchitis or asthma. CRP not needed for diagnosis.

***Acute bronchitis****:*

*Usual symptoms and findings:*

Scattered bilateral crepitations, coloured sputum and mild obstructiveness. The cough is often troublesome and lasts on average 3 weeks.

*Treatment:*

Antibiotics have no effect regardless of genesis (bacteria, virus or mycoplasma). Give written information about the natural course, available for example at the Public Health Authority website.

***Pneumonia:***

Affected patient who often has tachypnoea >20/min or tachycardia >120/min and symptoms/findings as follows:

*Common symptoms:*

Fever, cough, dyspnoea, recent pronounced tiredness and breathing-correlated chest pain.

*Common findings:*

Focally diminished breathing sound, focal crepitations (rales/rhonci) or dull sound under percussion.

CRP is not necessary in clinically clear pneumonia, but can be of value for following the course. Lung X-ray usually not necessary for diagnosis.

*Antibiotic treatment adults:*

Assess the seriousness to decide the level of therapy.

First-hand choice is PcV 1 g x 3 for 7 days. Initial coverage against mycoplasma is not necessary since self-healing is high. If treatment fails or in the event of penicillin allergy give doxycyklin for 7 days, 200 mg x 1 days 1–3, thereafter 100 mg x 1.

*Follow-up:*

Clinical check-up (telephone or visit) after 6–8 weeks.

***Uncertain lower respiratory tract infection:***

If the clinical picture is less clear, for example with cough, fever, subjective breathing difficulties and some constitutional symptoms, CRP can give guidance but must be assessed in relation to the duration of the disease.

CRP > 100 mg/L + clinical indications of pneumonia, consider antibiotics

CRP < 20 mg/L after > 24 hours rules out pneumonia with high probability, refrain from antibiotics

Symptoms > 1 week + CRP > 50 suggests pneumonia, consider antibiotics

*Treatment if uncertainty persists*:

In the first instance active wait and see. Consider lung X-ray or “prescription in reserve”.

**Pharyngotonsillitis**

*Background:*

Pharyngotonsillitis in just under half of cases is caused by beta-haemolytic streptococci group A, GAS. The majority of these patients become free of symptoms within a week whether antibiotics are given or not.

Patients with clear symptoms caused by streptococci can benefit from antibiotic treatment. Treatment can shorten the duration of symptoms by 1–2.5 days if the patient has findings of GAS and at least 3 of 4 Centor criteria.

The complication peritonsillitis occurs in roughly 2 per cent of all patients with pharyngotonsillitis. The risk can be reduced with antibiotic treatment. The complications rheumatic fever and glomerulonephritis are very rare and do not in themselves warrant antibiotic treatment. In the event of deviating symptoms or clinical status, consider other diagnoses, for example peritonsillitis.

*Diagnosis:*

Diagnosis is done in stages

1. Commons symptoms in a virally caused respiratory tract infection are, e.g., runny nose, cough, hoarseness or blisters in the mouth and throat. Taking samples for streptococci should be avoided.

2. In children under 3 throat infection caused by GAS is very uncommon and therefore another diagnosis should generally be considered.

3. Decide whether the advantages of antibiotic treatment outweigh the disadvantages for the individual before you decide on an aetiological diagnosis, e.g., quick test for GAS.

4. Use the Centor criteria to assess the need for GAS diagnosis for pharyngotonsillitis in patients ≥3 years:

•Fever ≥38.5 °C

•Tender adenitis in the jaw angle

•Coating on the tonsils*

•Lack of cough

* In children aged 3–6 years inflamed (red and swollen) tonsils are a sufficient criterion.

5. Quick test for GAS can be warranted if there are at least 3 Centor criteria. If there are fewer than 3 Centor criteria, no benefit has been shown for using antibiotics even if GAS is demonstrated.

6. If at least 3 Centor criteria are met and quick test for GAS is positive, antibiotic treatment is recommended. If quick test for GAS is negative, treatment to relieve symptoms is recommended as needed. Consider diagnosis for mononucleosis.

7. CRP cannot distinguish between bacterial and viral pharyngotonsillitis.

8. Inform the patient about the normal course of pharyngotonsillitis, irrespective of whether antibiotics are prescribed or not. New contact should be made in the event of deterioration or no improvement within 3 days.

*Antibiotic treatment adults and children:*

For antibiotic treatment the first choice is PcV.

Adults are given 1 g x 3 for 10 days, children are given 12.5 mg/kg body weight x 3 for 10 days.

If allergic to penicillin, prescribe klindamycin, especially if there is risk of type 1 reactions.

Adults are given 300 mg x 3 for 10 days, children are given 5 mg/kg body weight x 3 for 10 days.

In the event of recurrent infections, prescribe klindamycin (see dosage above), or cefadroxil.

Adults are given 500 mg x 2 for 10 days, children are given 15 mg/kg body weight x 2 for 10 days.
